# The most influential cephalometric factors affecting variations in the nasolabial angle: A cross-sectional regression study

**DOI:** 10.4317/jced.63552

**Published:** 2025-12-30

**Authors:** André Alexis Díaz-Quevedo, Luis Ernesto Arriola-Guillén

**Affiliations:** 1 Dentist, School of Dentistry, Universidad Científica del Sur, Lima, Perú. ORCID ID: 0000-0001-6156-2646; 2 Ph.D. and Associate Professor of the Division of Orthodontics, Universidad Científica del Sur, Lima, Perú. ORCID ID: 0000-0003-0010-5948

## Abstract

**Background:**

This study aims to evaluate the most influential cephalometric values affecting variations in the nasolabial angle among a sample of Peruvian individuals.

**Material and Methods:**

This retrospective cross-sectional study analyzed 111 lateral head radiographs from individuals aged 15 to 40 years for cephalometric evaluation. Two trained and calibrated researchers measured various cephalometric parameters, including the nasolabial angle, maxillary central incisor inclination (I-NA, UIPP), maxillary position (SNA, SNB, ANB), upper lip thickness (ULT), palatal plane (PP), and occlusal plane (OP) using specialized measurement software. Variables related to affiliation were also assessed. Shapiro-Wilk test and multiple linear regression analyses were performed with significance at p &lt; 0.05.

**Results:**

Age has a significant effect on the nasolabial angle (p = 0.017), with the angle increasing by 0.45° for each year of age. The position of the upper incisor (I-NA) also plays a significant role (p = 0.006); specifically, for every millimeter that the upper incisor moves forward, the nasolabial angle decreases by 1.94°. Additionally, the inclination of the upper incisor (UIPP) significantly influences the angle as well (p = 0.040), resulting in a decrease of 0.43° in the angle for every 1° increase in inclination. Furthermore, upper lip thickness (ULT) has a significant impact on the nasolabial angle (p = 0.002); with every millimeter increase in labial thickness, the nasolabial angle decreases by 1.57°.

**Conclusions:**

The nasolabial angle is primarily influenced by age, the position and inclination of the upper incisor, and the thickness of the upper lip. Orthodontists should consider this information when planning their treatments.

## Introduction

One of the purposes of orthodontic treatment is to restore dental aesthetics and the functionality of the stomatognathic system (1 , 2). Facial diagnosis including the facial profile is based on specific measurement parameters that guide the clinician in determining the degree of alteration in a patient's facial profile. Thus, one of the most used cephalometric measurements in orthodontic practice is the nasolabial angle, which directly influences the assessment of the harmony and aesthetics of a patient's facial profile (3 - 5). According to the literature, the ideal range for this angle is 90° to 120°; however, these values may vary depending on sex and ethnicity (6 - 9). This cephalometric angle should ideally remain within these normal ranges to achieve optimal results, (10) so it is essential to understand what other cephalometric factors may affect it. Currently, investigations are being conducted to evaluate the correlation of specific cephalometric parameters with the nasolabial angle (10 - 17). In this regard, some investigations concluded that the inclination of the maxillary incisor and the base of the nose showed a significant correlation with the nasolabial angle; However, the upper lip thickness parameter presented a weak correlation (10 , 11). Other studies have found correlations between the nasolabial angle and the position of the maxilla, (12 , 13) the palatal plane, (13) to a certain extent, the ANB angle, (14) or have even found no association between the nasolabial angle and the inclination of the maxillary incisor, (15 , 16) or the position of the jaws (17). However, in general, most studies evaluate the influence of cephalometric factors on the nasolabial angle through correlations and do not use mainly regressions that could identify and reveal the magnitude of the influence of these variables on this angle (10 - 17). The literature emphasizes the existence of compensation of cephalometric variables that modify the nasolabial angle; however, the evaluation does not indicate the magnitude with which each predictor variable may influence the nasolabial angle. Therefore, knowing which variables may have the most significant influence on this angle is necessary. This knowledge could benefit orthodontists by prioritizing certain cephalometric positions or characteristics during treatment planning, enabling them to improve and control nasolabial angle values, leading to satisfactory patient outcomes. Therefore, this study aimed to evaluate the most influential cephalometric values affecting variations in the nasolabial angle among a sample of Peruvian individuals.

## Material and Methods

**Figure 1:**
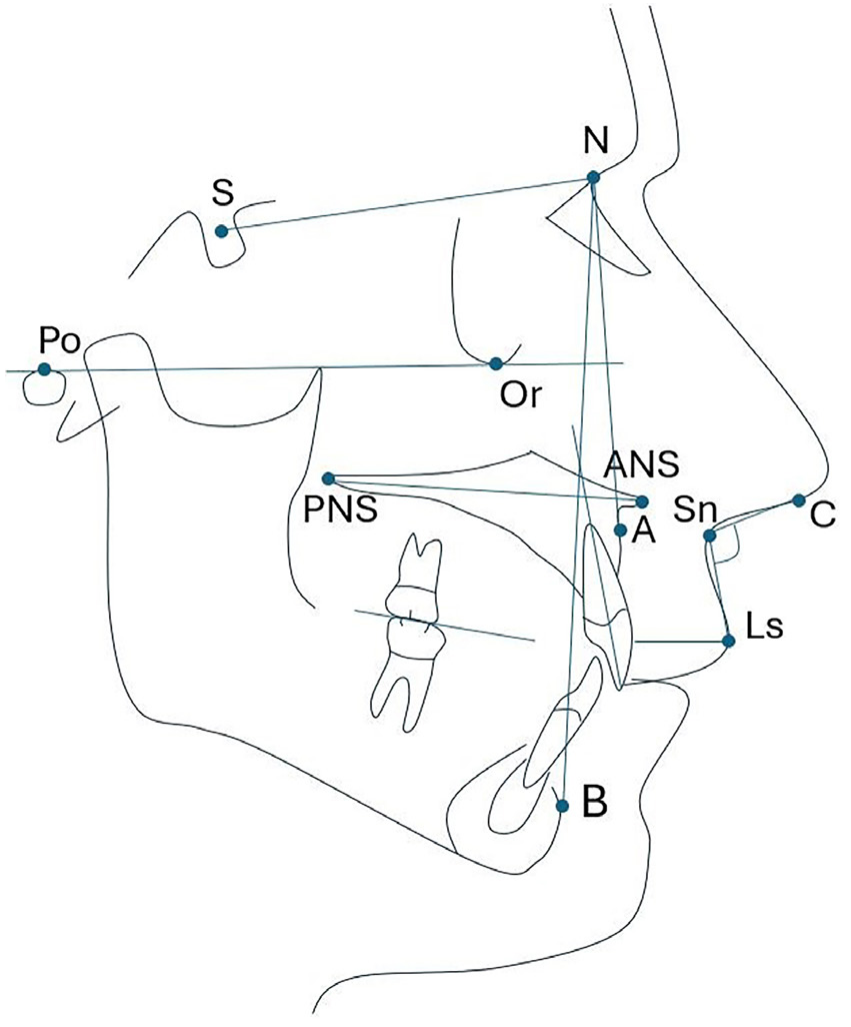
Illustration of the cephalometric features assessed in the sample studied.

This retrospective cross-sectional study adhered to the Strengthening the Reporting of Observational Studies in Epidemiology (STROBE) guidelines and the Declaration of Helsinki. It utilized a database of lateral head radiographs from individuals who visited radiology centers in Lima, Peru, between 2015 and 2020. This database was originally created for a previous study that received approval from the Ethics Committee of Científica del Sur University in Lima, Peru, under protocol number PRE-8-2022-00118. - Selection criteria Lateral head radiographs of individuals of both sexes, aged 15 to 40 years, were included in the study. These patients were attended in a radiology center in Lima, Peru for diagnostic imaging. Additionally, informed patient consent was required to use their X-rays in the study. Radiographs from individuals undergoing orthodontic treatment, those with craniofacial alterations, any tooth loss other than third molars, or those with unclear images were excluded from the study. - Sample size calculation The study included a total of 111 lateral head X-rays. The sample size calculation was conducted using Openepi.com software (https://www.openepi.com/SampleSize/SSPropor.htm), based on data gathered from a previous pilot test. The recorded parameters were as follows: Population (n=200), expected proportion (20%), confidence level (95%), test power (80%), and precision (5%). - Training and calibration An evaluator (AADQ) received training from an orthodontist (LEAG) with over 10 years of experience in performing cephalometric measurements. For calibration, 50 radiographs were measured twice over one week. Calibration values were obtained using the Dalbergh error method and measured with the intraclass correlation coefficient for quantitative variables. The correlation values for calibration were greater than 0.7 and less than 1° or 1mm to ensure acceptable measurement error. - Measurement of variables Cephalometric analysis of the lateral head radiographs was performed using BlueSky Plan 4 (USA). The images were converted into JPG format for the analysis of the following cephalometric measurements: nasolabial angle, maxillary central incisor inclination (I-NA, UIPP), maxillary jaw position (SNA, SNB, ANB), upper lip thickness (ULT), palatal plane (PP), and occlusal plane (OP) (Table 1, Fig. 1).

Table 1

1

- Statistical analysis Statistical analysis was conducted using SPSS version 24 (NY, USA). The variables were described using descriptive statistics, including mean, median, minimum and maximum values, and standard deviation. Data normality was assessed using the Shapiro-Wilk test. Multiple linear regression was performed to evaluate the influence of predictor variables (I-NA, ULT, ANB, PP) on the outcome variable (NLA). The "overfit" method involved performing an initial regression with all variables, then including those with a p-value &lt; 0.20 in a second regression to identify the variables with the most significant influence on the outcome. The significance level was set at p &lt; 0.05.

## Results

The initial characteristics of the sample, categorized by age and sex, are presented in Table 2.

Table 2

Table 3 presents the results of the multiple linear regression analysis, which investigated how various predictor variables affect the nasolabial angle.

Table 3

Variables with a p-value less than 0.020 were identified as having the most significant impact on the angle. In a second multiple linear regression analysis, these variables were evaluated using the overfit method. Table 4 demonstrates how the most predictive variables influence the nasolabial angle.

Table 4

It was observed that age significantly affects this angle (p = 0.017). Specifically, for each additional year of age, the nasolabial angle increases by 0.45° (upper labial flattening). Additionally, the position of the upper incisor (I-NA) also shows a significant influence (p = 0.006). Specifically, for every millimeter that the position of the upper incisor shifts forward, the nasolabial angle decreases by 1.94°, indicating an increase in upper labial protrusion. Furthermore, the inclination of the upper incisor (UIPP) significantly affects the angle as well (p = 0.040), with an increase of 1° in the upper incisor inclination relative to the palatal plane resulting in a decrease of 0.43° in the nasolabial angle (again reflecting upper labial protrusion). Lastly, the thickness of the upper lip significantly impacts the nasolabial angle (p = 0.002); for every millimeter increase in labial thickness, the nasolabial angle decreases by 1.57°, further contributing to upper labial protrusion.

## Discussion

In orthodontics, understanding the bony and dentoalveolar structures that can alter the position of the nasolabial angle is crucial. Orthodontists frequently modify this anatomical landmark, so they need to know which structures most significantly influence this aesthetic angle to achieve optimal results in treatment planning. Previous studies have examined the effects of various cephalometric values on the nasolabial angle (10 - 17). It has been observed that changes in dental position, particularly those involving the maxillary incisors, directly affect the nasolabial angle and, in turn, impact the aesthetics of the smile and facial profile (10 , 11). However, no studies have quantified the extent of influence that each cephalometric value has on the nasolabial angle. Therefore, the present study aimed to assess the specific effects of cephalometric values on the nasolabial angle. In this sense, our statistical analysis using multiple linear regression identified several key factors that significantly influence the nasolabial angle. These factors include age, the position and angle of inclination of the upper incisor, and the thickness of the upper lip. Specifically, we found that for each additional year of age, the nasolabial angle increases by 0.45°. This change indicates a flattening of the upper lip over time. This finding aligns with a study that compared three different age groups and concluded that the nasolabial angle increases as individuals age (18). Regarding cephalometric variables, upper incisor position (I-NA) we found an inverse relationship with the nasolabial angle, as each millimeter of forward movement of the upper incisor results in a 1.94° decrease in the angle. Likewise, if the angle of the upper incisor inclination relative to the palatal plane (UIPP) increases by 1°, it leads to a 0.43° decrease in the nasolabial angle. These findings imply that the configuration of the upper incisor can lead to greater upper lip protrusion, affecting the aesthetics of the facial profile (11). Additionally, the finding related to upper incisor inclination aligns with other studies suggesting a significant negative correlation between the incisor variable and the nasolabial angle (10 , 11). On the other hand, the thickness of the upper lip significantly affects the nasolabial angle. Specifically, a 1 mm increase in upper lip thickness results in a decrease of 1.57° in this angle. This indicates that a thicker lip contributes to greater lip protrusion, which has a notable aesthetic impact on the facial profile. In summary, the position of the incisors (I-NA) has the most significant impact on the nasolabial angle, with a decrease of 1.94° associated with changes in this factor. The second factor is upper lip thickness, which contributes to a reduction of 1.57°. Age also plays a role, positively influencing the angle by increasing it by 0.45°. Lastly, the inclination of the upper incisors (UIPP angle) affects the nasolabial angle, resulting in a decrease of 0.43°. Moreover, the study has some limitations, particularly because the sample was restricted to Peruvian patients. To address this, future research should include diverse population groups to yield more generalizable results. While many studies have shown that the nasolabial angle is influenced by various cephalometric variables, this research underscores the significance of each individual cephalometric value. This understanding can clarify how these values specifically affect the variability of the nasolabial angle and, in turn, facial aesthetics. These findings will greatly enhance the field of orthodontics by providing a more comprehensive and personalized approach to modifying the nasolabial angle based on each patient's unique characteristics. As a result, orthodontists will be better equipped to optimize the aesthetic outcomes of a patient's facial profile through more precise management of the nasolabial angle.

## Conclusions

In terms of influence on the nasolabial angle, the position of the incisors (I-NA) has the most significant impact, with a decrease of 1.94 ° associated with changes in this factor. This was followed by upper lip thickness, which contributes to a reduction of 1.57°, and age, which has a positive influence, increasing 0.45 °. Finally, the inclination of the upper incisors (UIPP angle) affects the nasolabial angle, resulting in a decrease of 0.43°. In brief, the nasolabial angle is inversely related to both the position of the upper incisors and the thickness of the upper lip; as these values increase, the nasolabial angle decreases. In contrast, age is directly related to the nasolabial angle, meaning that as age increases, the angle also increases.

## Figures and Tables

**Table 1:** Description of cephalometric values.

Value	Definition
Nasolabial angle	Angular value formed between points C-Sn, Sn-Ls.
SNA	Angular value formed between point Sella, Nasion, point A.
SNB	Angular value formed between point Sella, Nasion, point B.
ANB	Angular value formed between point A, Nasion, point B.
I-NA	Distance in millimeters from the incisal edge of the superior central incisor to the NA line.
INA	Angular value formed between the NA line and the longitudinal axis of the superior central incisor.
ULT	Thickness of the upper lip from Ls to the most convex point of the maxillary incisor.
UIPP	Angular value formed between the palatal plane (ANS-PNS) and the longitudinal axis of the upper central incisor.
PP	Distance in millimeters between the posterior nasal spine and the anterior nasal spine.
OP	Angular value formed between the Frankfort plane and the occlusion plane.

1

**Table 2:** Demographic characteristics of the sample (n=111).

Sex	n	Years		
		Mean	SD	p
Male	52	29.94	6.55	0.339
Female	59	28.90	4.85	

Student T test

**Table 3:** Influence of predictor variables on nasolabial angle.

Predictor variables	B	p	95.0% confidence interval for B	
			Lower limit	Upper limit
Sex	-2.106	0.505	-8.349	4.138
Age	0.423	0.031	0.039	0.807
I-NA (incisor position)	-2.026	0.005	-3.426	-0.626
I.NA (incisor inclination)	0.513	0.087	-0.077	1.102
UIPP angle (incisor inclination)	-0.313	0.177	-0.770	0.144
SNA angle	-2.596	0.328	-7.837	2.644
SNB angle	2.070	0.441	-3.244	7.385
ANB angle	2.593	0.337	-2.744	7.931
PNS-ANS (palatal plane length)	0.394	0.437	-0.607	1.395
Occlusal plane	0.112	0.701	-0.464	0.688
Upper lip thickness	-1.817	0.005	-3.057	-0.577

The highlighted values correspond to variables with a p-value < 0.200, which were considered for a new multiple linear regression analysis.

**Table 4:** Second multiple linear regression to evaluate the influence of predictor variables on the nasolabial angle.

Variables	B	p	95.0% confidence interval for B	
			Lower limit	Upper limit
Constant	145.18	<0.001*	96.21	194.15
Age	0.45	0.017*	0.08	0.82
I-NA (incisor position)	-1.94	0.006*	-3.30	-0.56
I.NA (incisor inclination)	0.54	0.053	-0.00	1.08
UIPP angle	-0.43	0.040*	-0.85	-0.20
Upper lip thickness	-1.57	0.002*	-2.52	-0.61

* Significant, R2 =27.9%

## Data Availability

The data supporting the findings of this study are available from the corresponding author upon reasonable request.
